# Dislocation Reduced by the Aid of the Ethereal Inhalation, with Other Notes

**Published:** 1847-04

**Authors:** C. H. Stedman


5. *Dislocation reduced by the aid of the ethereal inhalation, with other notices.*—James Murphy, a laboring man, aged 56, presented himself at the Hospital of the House of Correction on the 9th inst., having his left humerus dislocated into the axilla. The patient stated that the accident occurred five weeks ago, and that it was then seen by a physician of high respectability, who (no doubt by reason of the presence of inflammation and tumefaction) did not detect the true condition of the limb. The nature of the case was evident at the time of his coming here. This shoulder was less in size than the other, as was the whole arm for want of use. The elbow projected very considerably from the body, nor could the arm be rotated. The fingers were numb. The head of the bone could be distinctly felt in the axilla.

The operation for reduction was commenced by placing the patient on a bed. He then began to inspire through the ethereal inhaler. At this moment I observed that his knees were raised, and that there was much resistance of the muscles of the arm when slightly moved. I then, removing my boot, and sitting at his side, placed my heel in the axilla, and waited till the ether should have its expected effect. This occurred in about three minutes. His knees then relaxed and straightened, and as I gradually and firmly (with the assistance of a student) extended the arm and carried it a little further from the body, the head of the bone slipped into the socket. My own part in the operation was performed in less than two minutes. In a moment after the patient awoke from his lethargy, entirely unconscious of what had taken place.

On the afternoon of the same day I amputated the thumb of an old sailor while under the influence of the ethereal gas. He was totally unconscious of the operation, and said, when he awoke, that he had been dreaming that he was on board of a man of war in South America, walking the deck and chatting pleasantly with a shipmate.

A few weeks ago I amputated the leg of one man and the foot of another, while attempts were being made to render them insensible to pain by means of this same agent. But from want of docility in the patients, or from fear, or some other unaccountable influence, they failed to be affected by the gas to the desired extent. A very considerable mitigation of pain was, however, experienced by them, according to their own language.—C. H. Stedman, M. D., in *Ibid.*

